# Role of pharmacists in HIV prophylaxis: a scoping review of pharmacists’ services, interventions and outcomes

**DOI:** 10.1002/jia2.70090

**Published:** 2026-02-24

**Authors:** Gustavo Magno Baldin Tiguman, Amanda Veiga Barbosa das Dores, Patricia Melo Aguiar

**Affiliations:** ^1^ Department of Pharmacy Faculty of Pharmaceutical Sciences University of São Paulo São Paulo Brazil

**Keywords:** pharmacists, pharmaceutical services, pre‐exposure prophylaxis, post‐exposure prophylaxis, HIV prevention, health services research

## Abstract

**Introduction:**

HIV remains a major global public health challenge, with nearly 1.3 million new HIV acquisitions annually. Despite the effectiveness of pre‐exposure (PrEP) and post‐exposure prophylaxis (PEP), barriers to access, initiation and adherence persist. Pharmacists, as accessible healthcare providers with medication expertise, are increasingly involved in HIV prevention. However, the scope and impact of pharmacist‐led prophylaxis services have not been comprehensively synthesized. This study aimed to map and characterize the existing evidence on pharmacists’ services, interventions and outcomes in HIV prophylaxis.

**Methods:**

A scoping review was conducted with searches in Medline (PubMed), Embase, Scopus and LILACS, supplemented by grey literature (Google Scholar) until May 2025. Eligible studies included original research describing pharmacist‐led clinical activities or interventions related to HIV prophylaxis. Two independent reviewers conducted study selection and data extraction. Pharmacists’ roles were categorized based on the International Pharmaceutical Federation (FIP) and the Descriptive Elements of Pharmacist Intervention Characterization Tool (DEPICT) Version 2. The impacts of pharmacists on outcomes of care were categorized considering economic, clinical, humanistic and process‐related outcomes.

**Results:**

Out of 2736 records retrieved from searches, 26 studies published between 2014 and 2025 were included, which were conducted predominantly in the United States (*n* = 21). Pharmacists commonly performed direct patient care (e.g. patient counselling, PrEP/PEP prescribing, adherence monitoring, laboratory tests ordering), facilitated medication access and collaborated with other healthcare professionals via different communication methods (face‐to‐face, telephone, written, videoconference), especially in primary care and community pharmacies. Pharmacists’ autonomy to prescribe and order laboratory tests was observed in several studies under both independent and collaborative practice models. Twenty studies reported positive process outcomes, such as increased PrEP initiation, adherence and retention, while fewer assessed clinical (*n* = 8), economic (*n* = 2) or humanistic (*n* = 1) outcomes.

**Discussion:**

Expanding pharmacist‐led services, particularly in underserved regions, represents a promising strategy to improve access, initiation and adherence to HIV prevention. Future research on economic and patient‐centred outcomes is needed to inform integration of pharmacists into HIV prevention strategies.

**Conclusions:**

Pharmacists play a growing and multifaceted role in HIV prophylaxis across diverse healthcare settings. Evidence supports the expansion of pharmacist‐led models through enabling legislation, reimbursement policies and interprofessional collaboration.

## 1 INTRODUCTION

HIV remains a major global public health challenge, with approximately 39 million people living with HIV worldwide in 2023 and 1.3 million new HIV acquisitions occurring annually [1]. In 2021, nearly 718,000 deaths were attributed to HIV‐related causes globally, particularly in sub‐Saharan Africa [2]. While antiretroviral therapies have substantially decreased HIV‐related morbidity and mortality, prevention remains essential for reducing transmission [3]. Pharmacological prevention strategies, such as pre‐exposure prophylaxis (PrEP) and post‐exposure prophylaxis (PEP), are highly effective when implemented with appropriate prescriptions and sustained adherence [3, 4]. However, gaps in access, initiation and long‐term adherence limit their impact, particularly in underserved and resource‐limited settings [5, 6, 7].

Pharmacists are healthcare professionals uniquely positioned to bridge these gaps due to their accessibility, frequent patient contact and expertise in medication management [8]. Pharmacies are often the first point of healthcare access, especially in communities with limited availability of physicians and specialized HIV services [9, 10]. Beyond traditional roles in dispensing, pharmacists have increasingly been integrated into HIV prevention services, providing counselling, adherence support, laboratory monitoring, and in some jurisdictions, prescribing PrEP and PEP through collaborative practice agreements (CPAs) or legislative authorization [11, 12]. Evidence suggests that pharmacist‐led HIV prophylaxis services can reduce barriers to care, increase PrEP initiation and enhance patient retention [13, 14].

Global organizations, such as the International Pharmaceutical Federation (FIP), have emphasized the necessity of expanding pharmacist‐led public health services, including HIV prevention, as a pathway to optimizing healthcare systems [15]. Despite these advances, the role and impact of pharmacist‐led interventions in HIV prophylaxis have not been comprehensively summarized. Scoping reviews are particularly valuable in emerging areas of practice where evidence is heterogeneous and evolving. By systematically mapping existing research, these types of reviews can outline the wide range of pharmacist‐led activities, settings and reported outcomes, while identifying knowledge gaps to inform future research and policy [16].

Therefore, this scoping review aimed to synthesize the available evidence on the role of pharmacists in HIV prophylaxis, with a focus on clinical services and interventions for the provision of preventive care.

## 2 METHODS

### 2.1 Protocol and registration

A protocol detailing the methods and expected outcomes of this review was developed and stored on the Open Science Framework platform (https://osf.io/wyzem/). This scoping review was reported according to the recommendations of the Preferred Reporting Items for Systematic Reviews and Meta‐Analyses Statement for Scoping Reviews (PRISMA‐ScR) [17].

### 2.2 Eligibility criteria

The following research question was formulated for this scoping review: “What are the key clinical services and/or interventions performed by pharmacists in HIV prophylaxis?”. Original articles that described interventions performed by pharmacists with patients not living with HIV receiving prophylactic medications were included. Studies including mixed populations (people with HIV and individuals receiving HIV prophylaxis) were included only if pharmacist activities and outcomes related specifically to prophylactic medications could be clearly identified and extracted. The eligibility criteria used to select articles for inclusion were defined based on the PCC framework (Population [P]; Concept [C]; Context [C]), which was structured as follows:

- Population: Patients not living with HIV receiving prophylactic medications
- Concept: Clinical services and/or activities provided by pharmacists
- Context: All levels of healthcare services

We only included peer‐reviewed original studies; books, editorials, commentaries, conference abstracts, clinical guidelines and reviews (narrative, systematic, scoping, etc.) were not eligible. Articles unavailable in full‐text or published in non‐Roman characters (e.g. Japanese, Chinese, Arabic) were also excluded. No restrictions concerning the year of publication were applied.

### 2.3 Information sources and search strategy

A comprehensive literature search was conducted in Medline (via PubMed), Embase, Scopus, and Latin America and the Caribbean Health Sciences Literature (LILACS) on 20 May 2025. Grey literature searches were also performed in Google Scholar (up to the fifth page of results, excluding patents and citations) to identify potentially eligible articles not indexed in the above databases.

The search strategy combined the following Medical Subject Headings (MeSH) descriptors and their synonyms: “HIV,” “prophylaxis,” “pharmacists” and “pharmaceutical services.” The complete search strategy for all databases is available in Supplementary Material 1.

### 2.4 Study selection

Articles retrieved from database searches were exported to Rayyan QCRI, an online tool for study screening and selection (https://www.rayyan.ai/). Two researchers (GMBT and AVBD) independently reviewed titles and abstracts of studies from the searches; potentially eligible articles were then read in full‐text. Citations of selected articles were reviewed to identify relevant studies. Discrepancies in this process were resolved by involving a third reviewer (PMA).

### 2.5 Data extraction

The following data were extracted for each included article: first author, publication year, study conduct period, country where study was conducted, study design (case report, cross‐sectional, case‐control, prospective or retrospective cohort, quasi‐experimental, mixed‐methods, randomized controlled trial), population, sample size, type of prophylaxis (PrEP, PEP, other), clinical activities provided by pharmacists, outcomes (process, clinical, economic and humanistic) and main study results. Population categories were defined a priori based on the primary population targeted by each study; studies focusing exclusively on marginalized groups were categorized separately from general patient populations.

Clinical activities provided by pharmacists were categorized into three domains adapted from the definitions of the International Pharmaceutical Federation [18], considering the outpatient setting of HIV prophylaxis care: “Medication access and provision,” “Direct patient care” and “Support for other healthcare professionals.” The “Medication access and provision” dimension includes activities related to medication access, such as drug dispensing, support with patient access programmes, logistics and administrative functions. “Direct patient care” involves pharmacists providing patient counselling, obtaining medical and drug history, laboratory test ordering, monitoring adherence, performing pharmacotherapy review, identifying drug interactions, acting on adverse event reduction and prescribing medications such as PrEP and PEP. “Support for other healthcare professionals” includes activities performed by pharmacists in interdisciplinary teams, such as providing drug information to other professionals, referring patients to another specialty and providing interdisciplinary consultations.

Pharmacist clinical interventions were also characterized based on the main domains of the Descriptive Elements of Pharmacist Intervention Characterization Tool (DEPICT) Version 2: (1) recipient (patient, healthcare professional [HCP]); (2) recipient contact (one‐on‐one contact, group contact); (3) communication method with the recipient (face‐to‐face, written, telephone, videoconference); (4) intervention setting (community pharmacy, emergency department, hospital pharmacy, outpatient primary care setting, healthcare professional clinic); (5) actions taken by the pharmacist (structured educational programme, information about medications or patient counselling, notification about adherence, referral to another professional or service, changes or suggestion of changes in pharmacotherapy, request of laboratory tests, update of medication list, report of monitoring results); (6) materials supporting the actions (items developed or provided by pharmacists as part of their services); (7) medication therapy changes and laboratory tests (pharmacist autonomy to modify prescriptions or ordering of laboratory tests); and (8) prescribing model (with restrictions [dependent] or without restrictions [independent]) [19]. Dependent prescribing incorporates restrictions on prescribing activities via protocols or formularies by delegation of authority from an independent prescriber, usually a physician, involving a formal agreement. In an independent prescribing model, pharmacists can prescribe without the approval of another prescriber, that is, the pharmacist is solely responsible for prescribing [19].

The term “clinical activities” was used to broadly describe actions performed by pharmacists in HIV prophylaxis services according to the FIP definitions [18]. These activities are further characterized as “pharmacist clinical interventions” using the DEPICT Version 2 instrument, which operationalizes how such activities are delivered rather than defining a separate category of care [19]. The FIP framework and the DEPICT Version 2 instrument were used in a complementary manner: the former to classify pharmacist activities and services, and the latter to characterize the delivery and operational features of pharmacist clinical interventions [18, 19].

Outcomes were categorized into the following three domains according to the Economic, Clinical, Humanistic Outcomes (ECHO) model [20]: economic (e.g. reduction in healthcare costs), clinical (e.g. improved disease or symptom control) and humanistic (e.g. patient satisfaction and quality of life). The Donabedian's framework for process dimension was also used to characterize the impact of pharmacists in healthcare process optimization, which includes diagnosis, treatment, rehabilitation, prevention and patient education [21]. In this review, outcomes were considered “positive” in reference to favourable findings within predefined process, clinical, economic or humanistic outcome domains, rather than to a uniform or composite measure of effectiveness.

Two researchers independently extracted data using a preconfigured spreadsheet in Microsoft Excel 365 (GMBT and AVBD). Disagreements were resolved by a third researcher (PMA). A calibration test was conducted with two articles selected by the researchers to ensure consistency and alignment in the extraction process. During the calibration process, both reviewers independently extracted data from two selected articles and subsequently compared their results with the third researcher; discrepancies were discussed and resolved by consensus to refine and harmonize the data extraction process.

### 2.6 Synthesis of results

The results of this scoping review were presented through narrative and tabular synthesis. Given that the goal of scoping reviews is to identify all available evidence and summarize its main characteristics, regardless of quality, no methodological quality or risk of bias assessment was performed [22].

### 2.7 Ethics

Ethical approval and informed consent were not required for this study as it was a scoping review of publicly available literature and did not involve the collection or analysis of primary data from human participants.

## 3 RESULTS

### 3.1 Study selection

**Figure 1:**
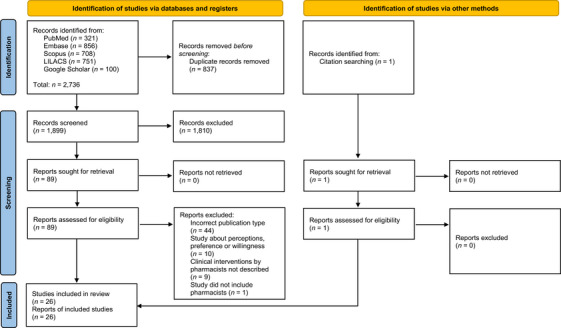
Flow diagram of the search, selection and inclusion process for eligible studies.

In total, 2736 records were identified from the selected databases. After removing duplicates, 1899 records were assessed for inclusion based on titles and abstracts, of which 89 were screened in full‐text against the inclusion criteria. Additionally, one report was identified from citation searching and was assessed for eligibility. In total, 26 studies were included in the review (Figure 1) [23, 24, 25, 26, 27, 28, 29, 30, 31, 32, 33, 34, 35, 36, 37, 38, 39, 40, 41, 42, 43, 44, 45, 46, 47, 48]. References from excluded studies with the reasons for their ineligibility are available in Supplementary Material 2.

### 3.2 Study characteristics

The characteristics of the studies included in the analysis are shown in Table 1. All 26 studies were published between 2014 and 2025 and the majority was conducted in the United States (*n* = 21) [23, 24, 25, 26, 27, 29, 30, 31, 32, 33, 34, 35, 36, 37, 38, 40, 41, 42, 44, 47, 48], although there were studies originated from Kenya (*n* = 2) [39, 43], Australia (*n* = 1) [28] and Nigeria (*n* = 1) [45]. Study designs varied between cross‐sectional (*n* = 8) [23, 24, 25, 29, 34, 38, 44, 45], case report (*n* = 6) [26, 27, 33, 35, 36], prospective cohort (*n* = 4) [31, 32, 39, 43], retrospective cohort (*n* = 4) [30, 37, 40, 47], quasi‐experimental (*n* = 2) [46, 48], mixed‐methods (*n* = 1) [41] and randomized controlled trial (*n* = 1) [28]. The target populations ranged between general patients eligible to receive prophylaxis (*n* = 15) [26, 27, 30, 31, 32, 33, 35, 36, 37, 39, 41, 43, 46, 47, 48], pharmacists (*n* = 8) [23, 24, 25, 28, 29, 34, 38, 45], veterans (*n* = 2) [42, 44] and individuals experiencing homelessness (*n* = 1) [40]. Three studies evaluated exclusively oral PEP [45, 47, 48] and two included both oral PrEP and PEP [33, 38] as prophylaxis types; all remaining studies evaluated the use of oral PrEP as prophylaxis (*n* = 21). No study reported pharmacist involvement in long‐acting injectable HIV prevention regimens.

**Table 1:** Characteristics of studies included in the scoping review (*N* = 26)

Authors (publication year)	Study period	Country	Study design	Population	Sample size	Type of HIV prophylaxis
Shaeer KM, et al. (2014) [23]	2014	United States	Cross‐sectional	Pharmacists	225	PrEP
Broekhuis JM, et al. (2018) [24]	2018	United States	Cross‐sectional	Pharmacists	140	PrEP
Okoro O and Hillman L. (2018) [25]	2018	United States	Cross‐sectional	Pharmacists	347	PrEP
Tung EL, et al. (2018) [26]	2018	United States	Case report	General patients eligible to receive prophylaxis	695	PrEP
Gauthier TP, et al. (2019) [27]	2019	United States	Case report	General patients eligible to receive prophylaxis	79	PrEP
Lal L, et al. (2019) [28]	2019	Australia	RTC	Pharmacists	12 pharmacies	PrEP
Meyerson BE, et al. (2019) [29]	2019	United States	Cross‐sectional	Pharmacists	284	PrEP
Hoth AB, et al. (2019) [30]	2019	United States	Retrospective cohort	General patients eligible to receive prophylaxis	186	PrEP
Havens JP, et al. (2019) [31]	2019	United States	Prospective cohort	General patients eligible to receive prophylaxis	60	PrEP
Khosropour CM, et al. (2020) [32]	2020	United States	Prospective cohort	General patients eligible to receive prophylaxis	69	PrEP
Lopez MI, et al. (2020) [33]	2020	United States	Case report	General patients eligible to receive prophylaxis	PrEP: 53; PEP: 6	PrEP and PEP
Tidd M, et al. (2022) [34]	2022	United States	Cross‐sectional	Pharmacists	26	PrEP
Miller TA, et al. (2022) [35]	2022	United States	Case report	General patients eligible to receive prophylaxis	76	PrEP
Lee LC, et al. (2023) [36]	2023	United States	Case report	General patients eligible to receive prophylaxis	122	PrEP
Greenwell K, et al. (2023) [37]	2023	United States	Retrospective cohort	General patients eligible to receive prophylaxis	149	PrEP
Hunter LA, et al. (2023) [38]	2023	United States	Cross‐sectional	Pharmacists	919	PrEP and PEP
Nakambale HN, et al. (2023) [39]	2023	Kenya	Prospective cohort	General patients eligible to receive prophylaxis	230	PrEP
McElyea J, et al. (2023) [40]	2023	United States	Retrospective cohort	Individuals experiencing homelessness	40	PrEP
Khosropour CM, et al. (2023) [41]	2023	United States	Mixed‐methods	General patients eligible to receive prophylaxis	121	PrEP
Cameron MA, et al. (2023) [42]	2023	United States	Case report	Veterans	53	PrEP
Omollo V, et al. (2023) [43]	2023	Kenya	Prospective cohort	General patients eligible to receive prophylaxis	287	PrEP
Kerbler MK, et al. (2024) [44]	2024	United States	Cross‐sectional	Veterans	26	PrEP
Isah, A, et al. (2024) [45]	2024	Nigeria	Cross‐sectional	Pharmacists	77	PEP
Zewdie KB, et al. (2024) [46]	2024	Kenya	Quasi‐experimental	General patients eligible to receive prophylaxis	746	PrEP
Brennan LM, et al. (2024) [47]	2024	United States	Retrospective cohort	General patients eligible to receive prophylaxis	52	PEP
Kaucher KA, et al. (2025) [48]	2025	United States	Quasi‐experimental	General patients eligible to receive prophylaxis	369	PEP

Abbreviations: NR, not reported; RTC, randomized controlled trial.

### 3.3 Clinical services and/or activities provided by pharmacists

Pharmacists provided a wide range of clinical services and/or activities in the included studies, as detailed in Table 2. The role of pharmacists in “Medication access and provision” (including drug dispensing, support with patient access programmes and administrative functions) was reported in 25 (96%) out of the 26 studies [23, 24, 25, 26, 27, 28, 29, 31, 32, 33, 34, 35, 36, 37, 38, 39, 40, 41, 42, 43, 44, 45, 46, 47, 48]. All of the 26 included studies reported activities related to “Direct patient care,” the majority of which involved patient counselling (*n* = 23) [23, 24, 25, 26, 27, 28, 29, 30, 31, 32, 33, 34, 35, 36, 37, 39, 40, 41, 42, 43, 46, 47, 48], pharmacist prescribing of PrEP and PEP (*n* = 15) [26, 27, 31, 32, 33, 35, 36, 37, 38, 39, 40, 41, 42, 43, 44], adherence monitoring (*n* = 15) [26, 27, 28, 30, 32, 33, 35, 36, 40, 41, 43, 45, 46, 47, 48], laboratory test ordering (*n* = 13) [26, 27, 30, 31, 32, 33, 35, 36, 37, 40, 41, 42, 43] and reduction of adverse events (*n* = 13) [26, 27, 32, 33, 35, 36, 40, 41, 43, 45, 46, 47, 48]. Twelve studies (46%) reported roles related to “Support to other healthcare professionals,” which included referral to another healthcare professional (*n* = 10) [26, 30, 31, 32, 33, 35, 41, 42, 43, 47], provision of drug information to other healthcare professionals (*n* = 3) [25, 30, 44] and multidisciplinary consultations (*n* = 1) [43].

**Table 2:** Clinical services and/or activities provided by pharmacists described in the studies included in the scoping review, based on the adapted domains from the International Pharmaceutical Federation (*N* = 26)

Authors (publication year)	Medication access and provision	Direct patient care	Support for other healthcare professionals
Shaeer KM, et al. (2014) [23]	Dispensing	Patient counselling	NR
Broekhuis JM, et al. (2018) [24]	Dispensing	Patient counselling	NR
Okoro O and Hillman L. (2018) [25]	Dispensing	Patient counselling	Drug information to other healthcare professionals
Tung EL, et al. (2018) [26]	Dispensing, support with patient access programmes	Patient counselling, medical history, laboratory test ordering, adherence monitoring, reduction of AEs, pharmacist prescribing	Referral to another healthcare professional
Gauthier TP, et al. (2019) [27]	Administrative functions	Patient counselling, medical history, laboratory test ordering, adherence monitoring, reduction of AEs, drug interaction assessment, pharmacist prescribing	NR
Lal L, et al. (2019) [28]	Dispensing, support with patient access programmes, logistics, administrative functions	Patient counselling, adherence monitoring	NR
Meyerson BE, et al. (2019) [29]	Dispensing	Patient counselling	NR
Hoth AB, et al. (2019) [30]	NR	Patient counselling, laboratory test ordering, adherence monitoring	Drug information to other healthcare professionals, referral to another healthcare professional
Havens JP, et al. (2019) [31]	Dispensing	Patient counselling, medical history, laboratory test ordering, pharmacist prescribing	Referral to another healthcare professional
Khosropour CM, et al. (2020) [32]	Dispensing, support with patient access programmes	Patient counselling, medical history, laboratory test ordering, adherence monitoring, reduction of AEs, pharmacist prescribing	Referral to another healthcare professional
Lopez MI, et al. (2020) [33]	Dispensing	Patient counselling, medical history, laboratory test ordering, adherence monitoring, reduction of AEs, pharmacist prescribing	Referral to another healthcare professional
Tidd M, et al. (2022) [34]	Dispensing	Patient counselling	NR
Miller TA, et al. (2022) [35]	Dispensing, support with patient access programmes	Patient counselling, medical history, laboratory test ordering, adherence monitoring, reduction of AEs, pharmacist prescribing	Referral to another healthcare professional
Lee LC, et al. (2023) [36]	Dispensing, support with patient access programmes, administrative functions	Patient counselling, laboratory test ordering, adherence monitoring, reduction of AEs, pharmacist prescribing	NR
Greenwell K, et al. (2023) [37]	Dispensing	Patient counselling, laboratory test ordering, pharmacist prescribing	NR
Hunter LA, et al. (2023) [38]	Dispensing	Pharmacist prescribing	NR
Nakambale HN, et al. (2023) [39]	Dispensing	Patient counselling, medical history, pharmacist prescribing	NR
McElyea J, et al. (2023) [40]	Dispensing, support with patient access programmes	Patient counselling, laboratory test ordering, adherence monitoring, reduction of AEs, pharmacist prescribing	NR
Khosropour CM, et al. (2023) [41]	Dispensing, support with patient access programmes	Patient counselling, medical history, laboratory test ordering, adherence monitoring, reduction of AEs, pharmacist prescribing	Referral to another healthcare professional
Cameron MA, et al. (2023) [42]	Dispensing	Patient counselling, medical history, laboratory test ordering, pharmacist prescribing	Referral to another healthcare professional
Omollo V, et al. (2023) [43]	Dispensing	Patient counselling, medical history, laboratory test ordering, adherence monitoring, reduction of AEs, pharmacist prescribing	Referral to another healthcare professional, multidisciplinary consultation
Kerbler MK, et al. (2024) [44]	Administrative functions	Pharmacist prescribing	Drug information to other healthcare professionals
Isah, A, et al. (2024) [45]	Dispensing	Adherence monitoring, reduction of AEs, drug interaction assessment, pharmacotherapy review	NR
Zewdie KB, et al. (2024) [46]	Dispensing	Patient counselling, medical history, adherence monitoring, reduction of AEs, pharmacotherapy review	NR
Brennan LM, et al. (2024) [47]	Dispensing	Patient counselling, adherence monitoring, reduction of AEs, drug interaction assessment, pharmacotherapy review	Referral to another healthcare professional
Kaucher KA, et al. (2025) [48]	Dispensing, support with patient access programmes, administrative functions	Patient counselling, adherence monitoring, reduction of AEs	NR
Total of studies (%)	25 (96%)	26 (100%)	12 (46%)

Abbreviations: AEs, adverse events; NR, not reported.

### 3.4 Characteristics of pharmacist interventions

The assessment of pharmacist interventions based on the key domains of the DEPICT Version 2 instrument (Table 3) demonstrated that recipients were patients (*n* = 16) [24, 26, 27, 28, 29, 31, 32, 33, 34, 36, 37, 38, 40, 41, 46, 48] and patients and HCPs (*n* = 10) [23, 25, 30, 35, 39, 42, 43, 44, 45, 47], whose contacts were mostly individual (one‐on‐one; *n* = 18) [26, 27, 30, 31, 32, 33, 35, 36, 37, 39, 40, 41, 42, 43, 44, 46, 47, 48]. In one study [42], contact with HCPs was made for a group of professionals. Different methods of communication were reported, such as face‐to‐face (*n* = 16) [26, 27, 31, 32, 33, 35, 36, 37, 39, 40, 41, 42, 43, 46, 47, 48], telephone (*n* = 12) [26, 27, 35, 36, 37, 39, 40, 41, 42, 43, 44], written (*n* = 7) [26, 35, 39, 41, 42, 43, 44] and videoconference (*n* = 3) [30, 36, 42]. Studies reported different interventions settings, including community pharmacy (*n* = 9) [25, 26, 28, 29, 31, 33, 34, 39, 43], ambulatory/primary care setting (*n* = 9) [30, 31, 32, 35, 36, 37, 40, 41, 46], HCP office (*n* = 2) [26, 42], Veterans Health Administration clinic (*n* = 2) [27, 44], hospital pharmacy (*n* = 1) [45], specialty pharmacy (*n* = 1) [47], emergency department (*n* = 1) [48] and clinical trial pharmacy (*n* = 1) [28]. In the included studies, pharmacists provided drug information or patient counselling (*n* = 23) [23, 24, 25, 26, 27, 28, 29, 30, 31, 32, 33, 34, 35, 36, 37, 39, 40, 41, 42, 43, 46, 47, 48], change or suggestion for change in therapy (*n* = 17) [26, 27, 31, 32, 33, 35, 36, 37, 38, 39, 40, 41, 42, 43, 44, 45, 47], lab tests order (*n* = 14) [26, 27, 30, 31, 32, 33, 35, 36, 37, 39, 40, 41, 42, 43], reminders/notification about non‐compliance (*n* = 14) [26, 27, 28, 30, 32, 33, 35, 36, 37, 40, 43, 45, 46, 48], monitoring results report (*n* = 10) [26, 27, 31, 32, 33, 35, 36, 40, 42, 48] and referral to other HCP or service (*n* = 10) [26, 30, 31, 32, 33, 35, 41, 42, 43, 47]. With regards to materials that supported actions taken by pharmacists, written action plan (*n* = 2) [26, 33], reminders (*n* = 2) [36, 44], medication compliance device (*n* = 1) [26] and pictorial instructions (*n* = 1) [46] were identified. Pharmacists had autonomy to start prescription medication in 15 studies [26, 27, 31, 33, 35, 36, 37, 38, 39, 40, 41, 43, 44] and autonomy to order laboratory tests in 13 studies [26, 27, 30, 31, 32, 33, 35, 36, 37, 39, 40, 42, 43]. Two studies reported pharmacists’ autonomy to suspend and/or change prescription medication [32, 42]. Dependent prescribing models (with restrictions) were found in nine studies [26, 30, 31, 32, 33, 35, 36, 40, 41], while independent prescribing models (without restrictions) were identified in seven studies [27, 37, 38, 39, 42, 43, 44].

**Table 3:** Characterization of pharmacist interventions based on the key domains of the DEPICT Version 2 (*N* = 26)

Authors (publication year)	Recipient	Contact with recipient	Methods of communication	Intervention setting	Action(s) taken by pharmacist	Materials that supported action(s)	Changes in medication therapy and laboratory tests	Prescribing model
Shaeer KM, et al. (2014) [23]	Patient and HCP	NR	NR	NR	Drug information or patient counselling (patient and HCP)	NR	NA	NA
Broekhuis JM, et al. (2018) [24]	Patient	NR	NR	NR	Drug information or patient counselling	NR	NA	NA
Okoro O and Hillman L. (2018) [25]	Patient and HCP	NR	NR	Community pharmacy (patient), NR (HCP)	Drug information or patient counselling (patient and HCP)	NR	NA	NA
Tung EL, et al. (2018) [26]	Patient	One‐on‐one	Face‐to‐face, telephone, written	Community pharmacy, HCP office	Drug information or patient counselling, reminders/notification about non‐compliance, referral to other HCP or service, lab tests order, change or suggestion for change in therapy, monitoring results report	Medication compliance device, written action plan	Autonomy to start prescription medication, autonomy to order laboratory tests	Dependent (with restrictions)
Gauthier TP, et al. (2019) [27]	Patient	One‐on‐one	Face‐to‐face, telephone	Veterans Health Administration clinic	Drug information or patient counselling, reminders/notification about non‐compliance, lab tests order, change or suggestion for change in therapy, monitoring results report	NR	Autonomy to start prescription medication, autonomy to order laboratory tests	Independent (without restrictions)
Lal L, et al. (2019) [28]	Patient	NR	NR	Community pharmacy and clinical trial pharmacy	Drug information or patient counselling, reminders/notification about non‐compliance	NR	NA	NA
Meyerson BE, et al. (2019) [29]	Patient	NR	NR	Community pharmacy	Drug information or patient counselling	NR	NA	NA
Hoth AB, et al. (2019) [30]	Patient and HCP	One‐on‐one (patient and HCP)	Videoconference (patient), NR (HCP)	Ambulatory/primary care setting (patient and HCP)	Drug information or patient counselling, reminders/notification about non‐compliance, referral to other HCP or service, lab tests order	NR	Autonomy to order laboratory tests	Dependent (with restrictions)
Havens JP, et al. (2019) [31]	Patient	One‐on‐one	Face‐to‐face	Community pharmacy, ambulatory/primary care setting	Drug information or patient counselling, referral to other HCP or service, lab tests order, change or suggestion for change in therapy, monitoring results report	NR	Autonomy to start prescription medication, autonomy to order laboratory tests	Dependent (with restrictions)
Khosropour CM, et al. (2020) [32]	Patient	One‐on‐one	Face‐to‐face	Ambulatory/primary care setting	Drug information or patient counselling, reminders/notification about non‐compliance, referral to other HCP or service, lab tests order, change or suggestion for change in therapy, monitoring results report	NR	Autonomy to start, suspend or change prescription medication, autonomy to order laboratory tests	Dependent (with restrictions)
Lopez MI, et al. (2020) [33]	Patient	One‐on‐one	Face‐to‐face	Community pharmacy	Drug information or patient counselling, reminders/notification about non‐compliance, referral to other HCP or service, lab tests order, change or suggestion for change in therapy, monitoring results report	Written action plan	Autonomy to start prescription medication, autonomy to order laboratory tests	Dependent (with restrictions)
Tidd M, et al. (2022) [34]	Patient	NR	NR	Community pharmacy	Drug information or patient counselling	NR	NA	NA
Miller TA, et al. (2022) [35]	Patient and HCP	One‐on‐one (patient and HCP)	Face‐to‐face, telephone, written (patient), NR (HCP)	Ambulatory/primary care setting (patient and HCP)	Drug information or patient counselling, reminders/notification about non‐compliance, referral to other HCP or service, lab tests order, change or suggestion for change in therapy, monitoring results report	NR	Autonomy to start prescription medication, autonomy to order laboratory tests	Dependent (with restrictions)
Lee LC, et al. (2023) [36]	Patient	One‐on‐one	Face‐to‐face, telephone, videoconference	Ambulatory/primary care setting	Drug information or patient counselling, reminders/notification about non‐compliance, lab tests order, change or suggestion for change in therapy, monitoring results report	Reminders	Autonomy to start prescription medication, autonomy to order laboratory tests	Dependent (with restrictions)
Greenwell K, et al. (2023) [37]	Patient	One‐on‐one	Face‐to‐face, telephone	Ambulatory/primary care setting	Drug information or patient counselling, reminders/notification about non‐compliance, lab tests order, change or suggestion for change in therapy	NR	Autonomy to start prescription medication, autonomy to order laboratory tests	Independent (without restrictions)
Hunter LA, et al. (2023) [38]	Patient	NR	NR	NR	Change or suggestion for change in therapy	NR	Autonomy to start prescription medication	Independent (without restrictions)
Nakambale HN, et al. (2023) [39]	Patient and HCP	One‐on‐one (patient and HCP)	Face‐to‐face (patient), written and telephone (HCP)	Community pharmacy (patient and HCP)	Drug information or patient counselling, lab tests order, change or suggestion for change in therapy	NR	Autonomy to start prescription medication, autonomy to order laboratory tests	Independent (without restrictions)
McElyea J, et al. (2023) [40]	Patient	One‐on‐one	Face‐to‐face, telephone	Ambulatory/primary care setting	Drug information or patient counselling, reminders/notification about non‐compliance, lab tests order, change or suggestion for change in therapy, monitoring results report	NR	Autonomy to start prescription medication, autonomy to order laboratory tests	Dependent (with restrictions)
Khosropour CM, et al. (2023) [41]	Patient	One‐on‐one	Face‐to‐face, telephone, written	Ambulatory/primary care setting	Drug information or patient counselling, referral to other HCP or service, lab tests order, change or suggestion for change in therapy	NR	Autonomy to start prescription medication	Dependent (with restrictions)
Cameron MA, et al. (2023) [42]	Patient and HCP	One‐on‐one (patient) and group (HCP)	Face‐to‐face, telephone and videoconference (patient), face‐to‐face and written (HCP)	HCP office (patient and HCP)	Drug information or patient counselling, referral to other HCP or service, lab tests order, change or suggestion for change in therapy, monitoring results report	NR	Autonomy to start, suspend or change prescription medication, autonomy to order laboratory tests	Independent (without restrictions)
Omollo V, et al. (2023) [43]	Patient and HCP	One‐on‐one (patient and HCP)	Face‐to‐face (patient), telephone and written (HCP)	Community pharmacy (patient and HCP)	Drug information or patient counselling, reminders/notification about non‐compliance, referral to other HCP or service, lab tests order, change or suggestion for change in therapy	NR	Autonomy to start prescription medication, autonomy to order laboratory tests	Independent (without restrictions)
Kerbler MK, et al. (2024) [44]	Patient and HCP	One‐on‐one (patient and HCP)	Telephone (patient), telephone and written (HCP)	Veterans Health Administration clinic (patient and HCP)	Change or suggestion for change in therapy	Reminders	Autonomy to start prescription medication	Independent (without restrictions)
Isah, A, et al. (2024) [45]	Patient and HCP	NR	NR	Hospital pharmacy (patient and HC)	Reminders/notification about non‐compliance, change or suggestion for change in therapy (patient and HCP)	NR	NA	NA
Zewdie KB, et al. (2024) [46]	Patient	One‐on‐one	Face‐to‐face	Ambulatory/primary care setting	Drug information or patient counselling, reminders/notification about non‐compliance	Pictorial instructions	NA	NA
Brennan LM, et al. (2024) [47]	Patient and HCP	One‐on‐one (patient and HCP)	Face‐to‐face (patient), NR (HCP)	Specialty pharmacy (patient and HCP)	Drug information or patient counselling, referral to other HCP or service, change or suggestion for change in therapy	NR	NA	NA
Kaucher KA, et al. (2025) [48]	Patient	One‐on‐one	Face‐to‐face, telephone	Emergency department	Drug information or patient counselling, reminders/notification about non‐compliance, monitoring results report	NR	NA	NA
Total of studies (%)	26 (100%)	18 (69%)	18 (69%)	23 (88%)	26 (100%)	5 (19%)	16 (62%)	16 (62%)

Abbreviations: HCP, healthcare professional; NA, not applicable; NR, not reported.

### 3.5 Impacts of pharmacists on outcomes of care in HIV prophylaxis

The impacts of pharmacists on outcomes of care in HIV prophylaxis are available in Table 4. Results from six [23, 24, 25, 29, 34, 38] were not included as they mainly aimed to investigate PrEP preferences or willingness among pharmacists rather than patient or process outcomes of care, even though they described pharmacist clinical activities or services. All of the 20 remaining studies reported improvements in process outcomes [26, 27, 28, 30, 31, 32, 33, 35, 36, 37, 39, 40, 41, 42, 43, 44, 45, 46, 47, 48], which included pharmacist impacts on PrEP initiation and adherence, identification of dispensation errors, patient retention, completion of laboratory tests, referral to another healthcare provider, patient counselling, identification of potentially eligible patients, time spent at clinics, among others. Clinical outcomes were described in eight studies [26, 27, 30, 31, 35, 37, 42, 48], which were mostly related to laboratory screening, seroconversion rates and frequency of adverse events. The minority of studies reported economic (*n* = 2) [26, 47] and humanistic (*n* = 1) [31] outcomes—these included analyses on cost savings for the acquisition of prophylactic medications among insured and uninsured individuals, as well as patient satisfaction and recommendation of the pharmacist‐led programmes.

**Table 4:** Impact of pharmacists on outcomes of care in HIV prophylaxis (*N* = 26)

Authors (publication year)	Process	Economic	Clinical	Humanistic
Tung EL, et al. (2018) [26]	In the community pharmacy‐based PrEP service, 90% of patients who received PrEP had a mean proportion of days covered greater than 80%.	98% of patients had a zero‐dollar patient responsibility per month, including uninsured individuals.	270 diagnoses of STIs were made. There were no HIV seroconversions in the service after the collaborative agreement was implemented.	
Gauthier TP, et al. (2019) [27]	The interdisciplinary PrEP model incorporated clinical pharmacist encounters into the structure and an antimicrobial stewardship programme oversight was implemented. In relation to retention in care, 87% of patients have had a follow‐up encounter with a healthcare professional 3 months after PrEP initiation. Of patients who remained on PrEP therapy after 12 months, 91% had 3 out of 4 quarters and 75% had 4 out of 4 PrEP visits, respectively.		One patient (1%) seroconverted while on PrEP, which was attributed to non‐adherence.	
Lal L, et al. (2019) [28]	2.0% of PrEP dispensations had an error, which were predominantly minor.			
Hoth AB, et al. (2019) [30]	Retention in the pharmacist‐led TelePrEP programme at 6 months was 61%, and 96% of indicated laboratory tests were completed. All patients diagnosed with STIs were referred to local treatment within 14 days (80% within 3 days).		Laboratory screening identified 37 STIs.	
Havens JP, et al. (2019) [31]	Patient retention in the pharmacist‐led PrEP programme at 3, 6, 9 and 12 months was 73%, 58%, 43% and 28%, respectively.		There were no HIV seroconversions in the service.	100% of participants who completed the patient satisfaction questionnaire would recommend the pharmacist‐led PrEP programme.
Khosropour CM, et al. (2020) [32]	All 69 patients evaluated by the pharmacist received a PrEP prescription; 77% filled their prescription. Of those, only 43% attended their initial clinical appointment within 6 weeks of obtaining the medication. There were no differences in PrEP initiation or retention by patient sex/gender.			
Lopez MI, et al. (2020) [33]	In the community pharmacy where PrEP and PEP services were offered, six patients received PEP and 53 completed a PrEP initiation visit, of whom 96% filled their prescription.			
Miller TA, et al. (2022) [35]	Of patients who engaged with the pharmacist‐led PrEP programme, 33.9% were retained in care for at least 1 year. Throughout programme implementation, areas of improvement were identified and addressed for process improvement.		There were no HIV seroconversions during pharmacist management programme.	
Lee LC, et al. (2023) [36]	Out of 29 patients with greater than a 90‐day PrEP supply, 10.3% were prescribed through the pharmacy CPA. None of the patients managed by the pharmacy CPA had overdue HIV testing.			
Greenwell K, et al. (2023) [37]	When comparing the pharmacist‐led telehealth clinic with the physician and nurse‐led in‐person clinic, adherence to PrEP medications and monitoring was similar between groups (PrEP tablets filled, serum creatinine screens and HIV screens per person‐year). However, patients were less likely to be lost to follow‐up in the pharmacist‐led telehealth clinic (11.9% vs. 30.0%; *p* = 0.009).		There were no new HIV seroconversions in neither group.	
Nakambale HN, et al. (2023) [39]	Pharmacy providers identified 425 patients as eligible for pharmacy‐delivered PrEP services and initiated 54% on PrEP. Out of 197 patients eligible for PrEP continuation, 63% refilled their medication.			
McElyea J, et al. (2023) [40]	After the implementation of a clinical pharmacist specialist programme, more patients experiencing homelessness were enrolled in patient assistance programmes (100% vs. 44%; *p*<0.01) and more patients picked up the first PrEP dispensation (80% vs. 40%; *p* = 0.04). The overall population had low dispensation rates and retention to care.			
Khosropour CM, et al. (2023) [41]	After the implementation of a pharmacist‐led, same‐day PrEP programme, one‐quarter (26%) of patients never filled their PrEP prescription, 44% picked up the prescription but never linked into clinical care, 12% linked into care at some point after 3 months (resulting in a gap in PrEP coverage) and 18% linked into care within 3 months.			
Cameron MA, et al. (2023) [42]	Clinical pharmacists provided direct patient care under a collaborative scope of practice and used novel telemedicine modes of care to improve access and patient acceptability. Considering all follow‐up visits, 20% of veterans stopped using PrEP. No PrEP discontinuations due to adverse reactions were observed.		No clinically significant adverse renal events were identified.	
Omollo V, et al. (2023) [43]	In total, 287 patients initiated PrEP through the pharmacy‐based delivery model, of whom 55% refilled the medication. At initiation, 99% of patients were counselled on PrEP adherence and 97% on potential side effects. All patients received HIV self‐testing before PrEP dispensing.			
Kerbler MK, et al. (2024) [44]	Pharmacists used an HIV PrEP dashboard and retrospective chart review to identify eligible patients for PrEP. Out of 26 eligible patients, 11.5% were enrolled and 26.9% declined PrEP.			
Isah, A, et al. (2024) [45]	In one of the participating hospitals, 5.8% of pharmacists changed patients’ PEP prescription without consulting the prescriber. Pharmacist clinical interventions in PEP management included those related to adverse drug reactions, drug interactions, comorbidities, adherence and dose adjustment, among others.			
Zewdie KB, et al. (2024) [46]	In this differentiated direct‐to‐pharmacy PrEP refill service supported with patient HIV self‐testing, patient navigator, and pharmacist‐led rapid risk assessment and dispensing, total time spent at the clinic was reduced by 35% compared with control clinics (median of 51 minutes at control vs. 33 minutes at intervention clinics; *p*<0.001), while time spent on HIV testing (20 vs. 20 minutes; *p* = 0.50) and pharmacy (8 vs. 8 minutes; *p* = 0.80) remained unchanged. PrEP continuation was higher at intervention versus control clinics: 45% versus 33% at month 1, 34% versus 25% at month 3 and 23% versus 16% at month 6. Adherence measured by the presence of PrEP in dried blood spots was also similar between study groups.			
Brennan LM, et al. (2024) [47]	In this specialty pharmacy, pharmacist counselling was offered to all patients, of whom 74.5% accepted it. Pharmacists made clinical interventions on 29.4% of PEP referrals, which included clarifications on quantity or selected medication, referrals that had refills on prescriptions and drug−drug interactions.	Patients receiving a PEP regimen of raltegravir + emtricitabine/tenofovir disoproxil fumarate (FTC/TDF) experienced cost savings of $1692.60 and $218.40 for those who were fully insured and uninsured, respectively. Patients who received PEP with dolutegravir + FTC/TDF had cost savings of $676.20 and $2725.50 for those insured and uninsured, respectively.		
Kaucher KA, et al. (2025) [48]	After the implementation of an emergency medicine pharmacist service involved in PEP counselling and dispensing, the number of patients completing at least one follow‐up HIV screening and the number of screening occurrences within 6 months of PEP initiation increased from 3.3% to 14% and 1.4% to 8.9%, respectively (*p*<0.01). During postimplementation, 40% of patients who completed a telephone survey reported completing the 28‐day PEP regimen.		Of patients who completed the postimplementation telephone survey, 70% experienced a PEP‐related adverse drug event.	
Total of studies (%)	20 (76.9%)	2 (7.7%)	8 (30.8%)	1 (3.8%)

*Note*: Outcomes are presented according to the ECHO model and Donabedian's process framework to ensure consistency across studies with heterogeneous outcome reporting.

Abbreviations: CPA, collaborative practice agreement; PEP, post‐exposure prophylaxis; PrEP, pre‐exposure prophylaxis; STI, sexually transmitted infection.

## 4 DISCUSSION

This scoping review synthesized the existing evidence on the role of pharmacists in HIV prophylaxis, mapping the range of clinical activities, services and interventions provided as well as the reported impacts on patient and process outcomes. Twenty‐six studies published between 2014 and 2025 were identified, with the majority being conducted in the United States and focusing primarily on oral PrEP. Nearly all included studies reported pharmacist roles in medication access and provision, reflecting their traditional dispensing function, but also highlighting their contribution to overcoming financial and administrative barriers through patient assistance programmes. Notably, all studies described the involvement of pharmacists in direct patient care, with patient counselling, pharmacist prescribing of PrEP and PEP, and medication adherence being the most common activities. A subset of studies also documented pharmacists’ contributions to interdisciplinary care, such as referrals and provision of drug information to other professionals. Most pharmacist clinical interventions were performed through face‐to‐face and telephone contacts, but written and virtual (videoconference) modalities were also employed, demonstrating the pharmacist's adaptability to different contexts to provide prophylactic care. Pharmacist‐led activities were predominantly described in the contexts of community pharmacies and ambulatory/primary care; however, a range of additional practice settings were also identified. Many studies reported that pharmacists had the autonomy to initiate prescriptions or order laboratory tests, operating under independent practice models or through collaborative dependent prescribing frameworks. In terms of outcomes, most studies reported pharmacists’ positive effects on process metrics—such as increased PrEP initiation, improved adherence, completion of required laboratory tests, retention in care, reduction of dispensing errors and identification of eligible individuals. Fewer studies reported clinical outcomes (e.g. seroconversion, reduction of adverse events, laboratory screening), and only a small number evaluated economic or humanistic outcomes (e.g. cost savings, patient satisfaction).

A significant number of studies on the role of pharmacists in HIV prophylaxis was originated in the United States. Pharmacist provision of HIV prophylaxis services is constantly evolving in this country, with the example of pharmacists’ prescribing authority for PrEP through legislation or CPAs in many states, resulting in increased access and use of PrEP [49, 50]. Such models may reduce delays in PrEP initiation and improve continuity of care, particularly in contexts where access to physicians is limited [49, 50]. While high‐income countries (HICs) present better structure and preparedness for the provision of pharmacist‐led HIV prophylaxis services, low‐ and middle‐income countries (LMICs) face several challenges related to infrastructure, funding and regulatory oversight that would require addressing policy gaps, strengthening pharmacist training and promoting patient‐centred approaches through collaborations between governments, health agencies and local communities [51]. Moreover, HICs have higher investments on pharmacy research in comparison to lower‐income economies, which leads to disparities in evidence generation to inform public policymaking and strengthen the pharmacy profession [52]. An example of the inequality in pharmacy practice and research is Brazil, where although pharmacist prescribing of PrEP and PEP is authorized following national clinical protocols [53], no Brazilian research investigation reporting pharmacist clinical activities or services was identified in this scoping review.

Our findings indicate that, although the majority of studies focused on the delivery of oral PrEP services, emerging evidence was also observed highlighting pharmacists’ involvement in oral PEP management. Previous reviews have similarly emphasized the predominance of PrEP‐related research within pharmacy practice, while excluding studies addressing PEP [12, 13, 54]. This imbalance suggests that, while pharmacists are increasingly engaged in HIV prevention through PrEP, their potential contributions to PEP services remain understudied. Further research is warranted to evaluate models of pharmacist integration into PEP provision, including aspects of accessibility, feasibility and outcomes of care. Moreover, although this scoping review did not encompass other HIV prevention methods due to the absence of explicit descriptions of pharmacist‐led clinical activities or services, pharmacists play important roles in several complementary prophylactic strategies, including participation in needle and syringe exchange programmes, patient education on condom use, HIV testing and linkage to care, and treatment‐as‐prevention initiatives, among others [55, 56, 57, 58, 59, 60, 61].

Although most pharmacist‐delivered HIV prophylaxis models identified in this review relied on daily oral PrEP regimens, the HIV prevention landscape is rapidly evolving towards longer‐acting modalities that may be particularly amenable to pharmacy‐based delivery [62]. Long‐acting injectable PrEP agents, such as cabotegravir and lenacapavir, and emerging infrequent‐dosing options shift prevention from daily pill‐based adherence towards visit‐based administration and longitudinal follow‐up, areas in which pharmacists are well positioned to contribute [63, 64, 65]. Given their accessibility, experience with injectable therapies and established role in medication monitoring, pharmacists could support long‐acting HIV prevention through appointment‐based administration, adherence tracking, laboratory coordination and management of missed doses [12]. However, successful adoption will depend on regulatory authority, reimbursement mechanisms and implementation research demonstrating clinical effectiveness, economic sustainability and patient acceptability of pharmacist‐supported long‐acting prophylaxis models [14].

An increasing number of studies have reported pharmacist‐led provision of HIV prophylaxis services facilitated by CPAs or independent prescribing models. In CPAs, prescribers delegate prescribing privileges and legally authorize pharmacists to initiate, modify or continue prophylactic medication under defined protocols [66]. In the context of PrEP and PEP, CPAs expand pharmacists’ autonomy to order and interpret laboratory tests, assess medication eligibility and prescribe prophylactic treatment—attributions that were previously restricted to physicians [12, 66]. Evidence from the United States indicate that the adoption of such agreements demonstrated improved access to PrEP through same‐day initiation, greater patient retention and increased prescribing rates in community pharmacy settings [49, 50]. CPAs also enable pharmacists to integrate prevention services into routine workflows while ensuring appropriate clinical oversight through physician collaboration, thereby maintaining patient safety and quality of care [12]. Despite these benefits, the implementation of CPAs remains highly variable across countries, and their absence in many LMICs limits the scalability of pharmacist‐led HIV prophylaxis models.

Despite the growing evidence supporting pharmacist‐led HIV prophylaxis services, the lack of formal provider status for pharmacists remains a critical barrier to the sustainability and scale‐up of these models. While CPAs enable pharmacists to initiate and manage PrEP and PEP under delegated authority, they do not address the underlying issue of reimbursement for clinical services [12, 14]. In many settings, pharmacist‐led HIV prevention programmes rely on institutional support, grant funding or indirect cost recovery, which limits their economic viability and broader implementation [12, 14]. Recognition of pharmacists as healthcare providers is essential to ensure sustainable reimbursement mechanisms that reflect their clinical contributions to HIV prevention [8, 67]. This challenge is especially relevant as HIV prevention strategies evolve towards more resource‐intensive models, including long‐acting injectable prophylaxis, which may require additional clinical time, infrastructure and follow‐up [13]. Addressing these barriers will require a shift towards implementation‐focused research that goes beyond proof‐of‐concept studies. Future investigations should prioritize implementation science approaches to evaluate adoption, fidelity, sustainability, cost‐effectiveness and policy feasibility of pharmacist‐led HIV prevention models [68]. Such evidence is essential to inform regulatory reform, reimbursement policy and health system integration, and to support pharmacists’ role as fully recognized providers in comprehensive HIV prevention strategies.

A considerable proportion of studies described clinical interventions directed primarily to patients, with a predominance of individual, face‐to‐face or telephone contacts. A previous scoping review on the impact of pharmacist‐led clinical services in patients with hypertension and hyperlipidaemia also found face‐to‐face and telephone‐based approaches, which were associated with higher medication adherence [69]. Another cluster randomized trial conducted in 53 community pharmacies in The Netherlands observed that individual pharmacist counselling by telephone at the start of therapy resulted in improved adherence in patients initiating the use of renin‐angiotensin system inhibitors [70]. Personalized pharmacist−patient interactions facilitate tailored risk reduction counselling, adherence reinforcement and early identification of adverse drug reactions, thereby improving prophylaxis effectiveness [13, 60, 71, 72]. Moreover, the growing inclusion of more novel remote communication modalities, such as videoconferencing, observed in some studies, reflects an evolution towards telepharmacy approaches that enhance access and continuity of care, particularly for populations facing geographical or stigma‐related barriers [73, 74, 75].

The predominance of studies conducted in community pharmacies suggest that these are strategic sites to increase accessibility to HIV prophylaxis. Community pharmacies are widely distributed across regions, including in underserved areas, and oftentimes have extended opening hours, which reduce geographic and temporal barriers to healthcare [54, 76, 77]. Pharmacies are frequently the first point of contact for individuals seeking healthcare, and when pharmacists are empowered to provide preventive services, patients experience reduced waiting times, increased likelihood of same‐day PrEP initiation and improved continuity of care [54, 76, 77, 78]. Additionally, pharmacist‐led services have been shown to improve medication adherence, facilitate ongoing monitoring and enhance patient satisfaction, underscoring their role as accessible and trusted providers within the healthcare system [54, 76, 77, 78, 79, 80]. Acceptability of pharmacist prescribing of PrEP is generally positive among pharmacists, but concerns remain about training, laboratory test ordering authority, reimbursement, workload and regulatory constraints [14, 81].

Pharmacist‐led clinical interventions were also commonly observed in ambulatory/primary care settings, where pharmacists often work in more integrated or interprofessional teams, allowing for closer collaboration with other healthcare providers. A systematic review about pharmacists’ involvement in interprofessional collaboration in primary care observed that pharmacist clinical services—such as medication review, patient interview and recommendations to the physician—were associated with improvements in blood pressure, diabetes control and dyslipidaemia [82]. This is a particularly relevant context as the provision of clinical pharmacy services in primary care enables laboratory screening, pharmacotherapy follow‐up and continuity of care, reducing the burden on the healthcare workforce through an interdisciplinary approach [83, 84].

The impact of pharmacist involvement was most frequently reported in process outcomes, including improvements in PrEP initiation, adherence, patient retention and laboratory monitoring. These outcomes are highly relevant to the effectiveness of HIV prophylaxis programmes, as successful PrEP use depends on timely initiation and sustained adherence [85]. Indeed, a scoping review conducted by Zhao et al. found that pharmacist‐led initiatives often aim to influence initiation, retention and adherence metrics, as these are more immediately measurable in implementation settings [13]. Our findings are corroborated by a previous systematic review that investigated the impact of clinical pharmacists on HIV outcomes, which indicated that pharmacist clinical interventions can improve antiretroviral adherence, reduce dispensing errors and enhance pharmacotherapy follow‐up [86].

Clinical outcomes, however, were less often reported (approximately 31% of studies) in this scoping review. When assessed, they primarily involved surrogate or intermediate markers, such as laboratory screening completion, seroconversion rates and adverse event monitoring. There is limited evidence on actual prevention efficacy or cost‐effectiveness data on pharmacist‐led prophylaxis models, and even fewer studies measuring patient satisfaction or health‐related quality of life in those settings. Cost savings (for insurers or health systems) and patient satisfaction/acceptability of pharmacist‐led models are vital to building the case for sustainable scale‐up. More robust evidence contemplating clinical, economic and humanistic outcomes are required to strengthen the role of pharmacists in HIV prophylaxis and address gaps and barriers for policy changes [12, 14].

The findings of this scoping review underpin the evolving role of pharmacists as accessible healthcare professionals capable of providing a wide variety of HIV prophylactic services in diverse settings [23, 24, 25, 26, 27, 28, 29, 30, 31, 32, 33, 34, 35, 36, 37, 38, 39, 40, 41, 42, 43, 44, 45, 46, 47, 48]. As demonstrated in this review, pharmacist authority to prescribe medications and order laboratory tests (either independently or through collaborative agreements) is a reality in many locations with promising results, especially in process and clinical outcomes. Policymakers should consider enabling regulations, reimbursement models and interprofessional training programmes that leverage pharmacists’ potential to expand equitable access to HIV prophylaxis services worldwide.

This scoping review highlights several limitations within the existing literature on pharmacist‐led HIV prophylaxis services. Most studies focused on oral PrEP, with limited evidence addressing PEP or emerging long‐acting prevention modalities. Economic and humanistic outcomes were infrequently assessed, and most studies originated from high‐income countries, particularly the United States, limiting generalizability to other healthcare contexts. These gaps underscore the need for future research evaluating diverse HIV prevention strategies, broader outcome domains and implementation in low‐ and middle‐income settings. This scoping review also has methodological limitations that should be acknowledged. Although multiple databases were searched, relevant databases such as Web of Science were not included. Conference abstracts and non–peer‐reviewed sources were excluded, which may have limited the identification of emerging evidence, particularly from countries with fewer peer‐reviewed publications. As the search was limited to studies published up to May 2025, the most recent researches may not have been captured in this rapidly evolving field. Finally, heterogeneity in study populations, settings and reported outcomes limited opportunities for direct comparisons between studies, although this is not the primary objective of a scoping review [16]. These gaps reflect opportunities for future research, particularly randomized trials and implementation science studies evaluating the real‐world impact of pharmacist interventions in different contexts.

## 5 CONCLUSIONS

This scoping review mapped and synthesized the available evidence on the role of pharmacists in HIV prophylaxis, revealing the growing involvement of these professionals across diverse healthcare settings. Pharmacists have demonstrated a wide scope of practice that extends beyond medication dispensing and interdisciplinary support to encompass direct patient care, including laboratory test ordering, adherence monitoring, and prescribing of PrEP and PEP under independent or collaborative practice models. Evidence consistently highlighted positive effects on process outcomes, such as improved PrEP initiation, adherence and retention in care, while data on clinical, economic and humanistic outcomes remain limited.

The findings underscore pharmacists’ capacity to address access gaps and strengthen HIV prevention services, particularly in underserved areas where healthcare resources are constrained. Expanding pharmacist‐led models through enabling legislation, reimbursement mechanisms and interprofessional collaboration may enhance the scalability and sustainability of HIV prophylaxis programmes globally. Future research should focus on generating robust evidence from controlled and longitudinal studies assessing efficacy, cost‐effectiveness and patient‐centred outcomes. Such studies are essential to inform policy development, optimize healthcare resource allocation and support the global goal of reducing new HIV acquisitions through comprehensive and accessible pharmacist‐led prevention strategies.

## COMPETING INTERESTS

The authors have no conflicts of interest to declare.

## AUTHOR CONTRIBUTIONS

GMBT participated in the study conception, acquisition, analysis and interpretation of data, and drafted the work. AVBD participated in the acquisition, analysis and interpretation of data, and critically reviewed the work for important intellectual content. PMA participated in the study conception, acquisition, analysis and interpretation of data, and critically reviewed the work for important intellectual content. All authors approve the final version of this manuscript and agree to be accountable for all aspects of the work in ensuring that questions related to the accuracy or integrity of any part of the work are appropriately investigated and resolved.

## Supporting information




**Supplementary Material 1**: Search strategies used in the consulted databases for the scoping review (searches conducted on May 2025).
**Supplementary Material 2**. Excluded records with their respective reasons for exclusion during the full‐text eligibility assessment.

## Data Availability

Data available on request from the authors.

## References

[1] World Health Organization . HIV statistics, globally and by WHO region, 2024. Geneva: UNAIDS/WHO; 2024 Available from: https://www.who.int/teams/global‐hiv‐hepatitis‐and‐stis‐programmes/hiv/strategic‐information/hiv‐data‐and‐statistics.

[2] Global Burden of Disease HIV Collaborators . Global, regional, and national burden of HIV/AIDS, 1990–2021, and forecasts to 2050, for 204 countries and territories: the Global Burden of Disease Study 2021. Lancet HIV. 2024;11(12):e807–e822. 39608393 10.1016/S2352-3018(24)00212-1 PMC11612058

[3] Mody A , Sohn AH , Iwuji C , Tan RKJ , Venter F , Geng EH . HIV epidemiology, prevention, treatment, and implementation strategies for public health. Lancet. 2024;403(10425):471–492. 38043552 10.1016/S0140-6736(23)01381-8

[4] Fonner VA , Dalglish SL , Kennedy CE , Baggaley R , O'Reilly KR , Koechlin FM , et al. Effectiveness and safety of oral HIV preexposure prophylaxis for all populations. AIDS. 2016;30(12):1973–1983. 27149090 10.1097/QAD.0000000000001145 PMC4949005

[5] Huang YA , Tao G , Smith DK , Hoover KW . Persistence with human immunodeficiency virus pre‐exposure prophylaxis in the United States, 2012–2017. Clin Infect Dis. 2021;72(3):379–385. 33527117 10.1093/cid/ciaa037

[6] Mayer KH , Allan‐Blitz LT . Post‐exposure prophylaxis to prevent HIV: new drugs, new approaches, and more questions. Lancet HIV. 2023;10(12):e816–e824. 37952551 10.1016/S2352-3018(23)00238-2 PMC11331403

[7] Mayer KH , Molina JM , Thompson MA , Anderson PL , Mounzer KC , De Wet JJ , et al. Emtricitabine and tenofovir alafenamide vs emtricitabine and tenofovir disoproxil fumarate for HIV pre‐exposure prophylaxis (DISCOVER): primary results from a randomised, double‐blind, multicentre, active‐controlled, phase 3, non‐inferiority trial. Lancet. 2020;396(10246):239–254. 32711800 10.1016/S0140-6736(20)31065-5 PMC9665936

[8] Walpola RL , Issakhany D , Gisev N , Hopkins RE . The accessibility of pharmacist prescribing and impacts on medicines access: a systematic review. Res Social Adm Pharm. 2024;20(5):475–486. 38326207 10.1016/j.sapharm.2024.01.006

[9] Hedima EW , Okoro RN . Primary health care roles of community pharmacists in low‐ and middle‐income countries: a mixed methods systematic review. BMC Health Serv Res. 2025;25(1):1269. 41034858 10.1186/s12913-025-13387-0 PMC12487115

[10] Figueira I , Teixeira I , Rodrigues AT , Gama A , Dias S . Point‐of‐care HIV and hepatitis screening in community pharmacies: a quantitative and qualitative study. Int J Clin Pharm. 2022;44(5):1158–1168. 36098836 10.1007/s11096-022-01444-1 PMC9469055

[11] d'Entremont‐Harris M , Ramsey TD , MacNabb K , Murphy A , Bishop A , Isenor JE , et al. Implementation and acceptance of pharmacists' prescribing of human immunodeficiency virus (HIV) pre‐exposure prophylaxis (PrEP). Can Pharm J (Ott). 2025;158(5):302–311. 40881659 10.1177/17151635251355277 PMC12373644

[12] Chandra C , Hudson AF , Alohan DI , Young HN , Crawford ND . Implementation science of integrating pre‐exposure prophylaxis in pharmacist‐led services in the United States. Curr HIV/AIDS Rep. 2024;21(4):197–207. 38775937 10.1007/s11904-024-00700-5 PMC11931410

[13] Zhao A , Dangerfield DT , Nunn A , Patel R , Farley JE , Ugoji CC , et al. Pharmacy‐based interventions to increase use of HIV pre‐exposure prophylaxis in the United States: a scoping review. AIDS Behav. 2022;26(5):1377–1392. 34669062 10.1007/s10461-021-03494-4 PMC8527816

[14] Harrison C , Family H , Kesten J , Denford S , Scott A , Dawson S , et al. Facilitators and barriers to community pharmacy PrEP delivery: a scoping review. J Int AIDS Soc. 2024;27(3):e26232. 38494652 10.1002/jia2.26232 PMC10945033

[15] International Pharmaceutical Federation (FIP) . Global situation report on pharmacy 2025: workforce, practice and policy. Hague: International Pharmaceutical Federation; 2025. Available from: https://www.fip.org/file/6352.

[16] Peters MD , Godfrey CM , Khalil H , McInerney P , Parker D , Soares CB . Guidance for conducting systematic scoping reviews. Int J Evid Based Healthc. 2015;13(3):141–146. 26134548 10.1097/XEB.0000000000000050

[17] Tricco AC , Lillie E , Zarin W , O'Brien KK , Colquhoun H , Levac D , et al. PRISMA Extension for Scoping Reviews (PRISMA‐ScR): checklist and explanation. Ann Intern Med. 2018;169(7):467–473. 30178033 10.7326/M18-0850

[18] International Pharmaceutical Federation (FIP) . Patient safety. Pharmacists' role in medication without harm. The Netherlands; 2020. Available from: https://www.fip.org/file/4757.

[19] Rotta I , Salgado TM , Felix DC , Souza TT , Correr CJ , Fernandez‐Llimos F . Ensuring consistent reporting of clinical pharmacy services to enhance reproducibility in practice: an improved version of DEPICT. J Eval Clin Pract. 2015;21(4):584–590. 25676042 10.1111/jep.12339

[20] Kozma CM . Outcomes research and pharmacy practice. Am Pharm. 1995;NS35(7):35–41. 10.1016/s0160-3450(16)33891-0 7661105

[21] Donabedian A , Bashshur R . An introduction to quality assurance in health care. New York: Oxford University Press; 2003.

[22] Peters MDJ , Marnie C , Colquhoun H , Garritty CM , Hempel S , Horsley T , et al. Scoping reviews: reinforcing and advancing the methodology and application. Syst Rev. 2021;10(1):263. 34625095 10.1186/s13643-021-01821-3 PMC8499488

[23] Shaeer KM , Sherman EM , Shafiq S , Hardigan P . Exploratory survey of Florida pharmacists' experience, knowledge, and perception of HIV pre‐exposure prophylaxis. J Am Pharm Assoc (Wash DC). 2014;54(6):610–617. 10.1331/JAPhA.2014.14014 25343624

[24] Broekhuis JM , Scarsi KK , Sayles HR , Klepser DG , Havens JP , Swindells S , et al. Midwest pharmacists' familiarity, experience, and willingness to provide pre‐exposure prophylaxis (PrEP) for HIV. PLoS One. 2018;13(11):e0207372. 30427912 10.1371/journal.pone.0207372 PMC6235377

[25] Okoro O , Hillman L . HIV pre‐exposure prophylaxis: exploring the potential for expanding the role of pharmacists in public health. J Am Pharm Assoc (Wash DC). 2018;58(4):412–420.e3. 10.1016/j.japh.2018.04.007 29789257

[26] Tung EL , Thomas A , Eichner A , Shalit P . Implementation of a community pharmacy‐based pre‐exposure prophylaxis service: a novel model for pre‐exposure prophylaxis care. Sex Health. 2018;15(6):556–561. 30401342 10.1071/SH18084

[27] Gauthier TP , Toro M , Carrasquillo MZ , Corentin M , Lichtenberger P . A PrEP model incorporating clinical pharmacist encounters and antimicrobial stewardship program oversight may improve retention in care. Clin Infect Dis. 2019;68(2):347–349. 10.1093/cid/ciy640 30107390

[28] Lal L , Ryan K , Yi Liu I , Price B , Lockwood T , Aguirre I , et al. Transformation of Australian community pharmacies into good clinical practice compliant trial pharmacies for HIV pre‐exposure prophylaxis. Front Pharmacol. 2019;10:1269. 31787893 10.3389/fphar.2019.01269 PMC6854879

[29] Meyerson BE , Dinh PC Jr , Agley JD , Hill BJ , Motley DN , Carter GA , et al. Predicting pharmacist dispensing practices and comfort related to pre‐exposure prophylaxis for HIV prevention (PrEP). AIDS Behav. 2019;23(7):1925–1938. 30607758 10.1007/s10461-018-02383-7 PMC8274484

[30] Hoth AB , Shafer C , Dillon DB , Mayer R , Walton G , Ohl ME . Iowa TelePrEP: a public‐health‐partnered telehealth model for human immunodeficiency virus preexposure prophylaxis delivery in a rural state. Sex Transm Dis. 2019;46(8):507–512. 31295217 10.1097/OLQ.0000000000001017

[31] Havens JP , Scarsi KK , Sayles H , Klepser DG , Swindells S , Bares SH . Acceptability and feasibility of a pharmacist‐led human immunodeficiency virus pre‐exposure prophylaxis program in the Midwestern United States. Open Forum Infect Dis. 2019;6(10):ofz365. 31412131 10.1093/ofid/ofz365 PMC6765348

[32] Khosropour CM , Backus KV , Means AR , Beauchamps L , Johnson K , Golden MR , et al. A pharmacist‐led, same‐day, HIV pre‐exposure prophylaxis initiation program to increase PrEP uptake and decrease time to PrEP initiation. AIDS Patient Care STDs. 2020;34(1):1–6. 31944854 10.1089/apc.2019.0235 PMC6983741

[33] Lopez MI , Cocohoba J , Cohen SE , Trainor N , Levy MM , Dong BJ . Implementation of pre‐exposure prophylaxis at a community pharmacy through a collaborative practice agreement with San Francisco Department of Public Health. J Am Pharm Assoc (Wash DC). 2020;60(1):138–144. 10.1016/j.japh.2019.06.021 31405804

[34] Tidd M , Shiyanbola O , Ford JH 2nd , Richert L . Assessing the use of an infographic on pre‐exposure prophylaxis for Wisconsin community pharmacists. J Am Pharm Assoc (2003). 2022;62(6):1897–1903 e4. 35989150 10.1016/j.japh.2022.07.010 PMC11008566

[35] Miller TA , Halza K , Hovis Z . Implementation of pharmacist‐led HIV pre‐exposure prophylaxis management to increase access to care at an academic internal medicine practice. J Am Coll Clin Pharm. 2022;5(9):988–994.

[36] Lee LC , Pollak BA , Coffey CP . Implementing a collaborative practice agreement for HIV pre‐exposure prophylaxis in the primary care setting. J Am Pharm Assoc (Wash DC). 2023;63(1):383–388. 10.1016/j.japh.2022.09.011 36244883

[37] Greenwell K , Fugit R , Nicholson L , Wright J . A retrospective comparison of HIV pre‐exposure prophylaxis (PrEP) outcomes between a pharmacist‐led telehealth clinic and in‐person clinic in a veteran population. AIDS Behav. 2023;27(11):3678–3686. 37247044 10.1007/s10461-023-04084-2 PMC10226437

[38] Hunter LA , Packel LJ , Chitle P , Beltran RM , Rafie S , De Martini L , et al. Opportunities to increase access to HIV prevention: evaluating the implementation of pharmacist‐initiated pre‐exposure prophylaxis in California. Open Forum Infect Dis. 2023;10(11):ofad549. 38023549 10.1093/ofid/ofad549 PMC10651201

[39] Nakambale HN , Roche SD , Mogere P , Omollo V , Kuo AP , Stergachis A , et al. Barriers to and strategies for early implementation of pharmacy‐delivered HIV PrEP services in Kenya: an analysis of routine data. Front Reprod Health. 2023;5:1023568. 36895656 10.3389/frph.2023.1023568 PMC9989195

[40] McElyea J , Bistransin K , Bana S , Alvarez KS , Brown LS , Persaud D , et al. Impact of a clinical pharmacist within an HIV PrEP program for patients experiencing homelessness. J Am Pharm Assoc (Wash DC). 2023;63(1):324–329. 10.1016/j.japh.2022.09.003 36184385

[41] Khosropour CM , Riley T , Healy E , Backus KV , Gomillia CE , Mena L , et al. Persistence in a pharmacist‐led, same‐day PrEP program in Mississippi: a mixed‐methods study. BMC Public Health. 2023;23(1):1130. 37312077 10.1186/s12889-023-16072-1 PMC10262591

[42] Cameron MA , Kawamoto J , Shahoumian TA , Belperio PS . Pharmacist‐led management of HIV PrEP within the Veterans Health Administration. Fed Pract. 2023;40(7):218–223. 37868711 10.12788/fp.0379 PMC10588999

[43] Omollo V , Asewe M , Mogere P , Maina G , Kuo AP , Odoyo J , et al. The fidelity of a pharmacy‐based oral HIV pre‐exposure prophylaxis delivery model in Kenya. J Acquir Immune Defic Syndr. 2023;93(5):379–386. 37079900 10.1097/QAI.0000000000003208 PMC10337311

[44] Kerbler MK , Isaacs C , Eatmon C , Reid J , Davis KW . Impact of an HIV pre‐exposure prophylaxis dashboard on veteran PrEP enrollment. J Am Pharm Assoc (Wash DC). 2024;64(2):471–475. 10.1016/j.japh.2024.01.002 38215824

[45] Isah A , Abubakar MM , Igboeli NU , Ugochukwu EJ , Aguiyi‐Ikeanyi CN , Akunne MO , et al. Pharmacists’ knowledge, attitude and practice of HIV post‐exposure prophylaxis: a cross‐sectional comparative study in two Nigerian teaching hospitals. Discover Public Health. 2024;21(1):237.

[46] Zewdie KB , Ngure K , Mwangi M , Mwangi D , Maina S , Etyang L , et al. Effect of differentiated direct‐to‐pharmacy PrEP refill visits supported with client HIV self‐testing on clinic visit time and early PrEP continuation. J Int AIDS Soc. 2024;27(3):e26222. 38446643 10.1002/jia2.26222 PMC10935714

[47] Brennan LM , Stickney KE , Allen GP , Springer S , Dube A , Bolduc C . Specialty pharmacy interventions benefit patients receiving HIV postexposure prophylaxis. J Am Pharm Assoc (2003). 2024;64(4):101915. 37696491 10.1016/j.japh.2023.09.001

[48] Kaucher KA , Acquisto NM , Gilliam E , Lowrey K , Metz M , Buchanan J . Improvement in HIV screening follow‐up rates with emergency medicine pharmacist counseling and dispensing of postexposure prophylaxis for sexual assault patients. JAPhA Pract Innov. 2025;2(1):100025.

[49] Fayaz‐Farkhad B . Expanding pharmacists’ prescribing authority and medication uptake: evidence from pre‐exposure prophylaxis. AJPM Focus. 2025;4(6):100415. 41141956 10.1016/j.focus.2025.100415 PMC12547906

[50] Le T , Kelly T . State policies on pharmacist‐initiated PrEP and PrEP usage. J Am Pharm Assoc (2003). 2025;65(5):102415. 40348187 10.1016/j.japh.2025.102415

[51] Lalla‐Edward ST , Venter WDF . Feasibility and impact of community pharmacy and novel pick‐up points for antiretroviral therapy pre‐exposure prophylaxis initiation and continuation in low and middle‐income countries. Curr HIV/AIDS Rep. 2024;22(1):2. 39548044 10.1007/s11904-024-00710-3 PMC11568023

[52] Abebe E . Positioning global pharmacy research partnerships to advance health equity. Res Social Adm Pharm. 2020;16(11):1619–1621. 32873525 10.1016/j.sapharm.2020.08.019 PMC7445131

[53] Tiguman GMB . Prescrição farmacêutica de profilaxia pré‐exposição e profilaxia pós‐exposição ao HIV (PrEP/PEP) no Brasil: regulamentação, cenário atual e perspectivas futuras. Brazil J Health Rev. 2024;7(9):e75644.

[54] Wang H , Guinn D , Ramisetty XR , Giordano TP , Poon IO . A review of studies on HIV pre‐exposure prophylaxis in community pharmacies in states with restrictive pharmacist prescription authority in the United States. Pharmacy (Basel). 2024;12(5):144. 39452799 10.3390/pharmacy12050144 PMC11510886

[55] Goodin A , Fallin‐Bennett A , Green T , Freeman PR . Pharmacists' role in harm reduction: a survey assessment of Kentucky community pharmacists' willingness to participate in syringe/needle exchange. Harm Reduct J. 2018;15(1):4. 29370808 10.1186/s12954-018-0211-4 PMC5785823

[56] Meyerson BE , Agley J , Crosby RA , Bentele KG , Vadiei N , Linde‐Krieger LB , et al. ASAP: a pharmacy‐level intervention to increase nonprescription syringe sales to reduce bloodborne illnesses. Res Social Adm Pharm. 2024;20(8):778–785. 38734511 10.1016/j.sapharm.2024.04.019 PMC11180557

[57] Kelly DV , Kielly J , Hughes C , Gahagan J , Asghari S , Hancock S , et al. Expanding access to HIV testing through Canadian community pharmacies: findings from the APPROACH study. BMC Public Health. 2020;20(1):639. 32380978 10.1186/s12889-020-08719-0 PMC7203868

[58] Darin KM , Klepser ME , Klepser DE , Klepser SA , Reeves A , Young M , et al. Pharmacist‐provided rapid HIV testing in two community pharmacies. J Am Pharm Assoc (2003). 2015;55(1):81–88. 25415222 10.1331/JAPhA.2015.14070

[59] Tkachuk S , Ready E , Chan S , Hawkes J , Janzen Cheney T , Kapler J , et al. Role of the pharmacist caring for people at risk of or living with HIV in Canada. Can Pharm J (Ott). 2024;157(5):218–239. 39310805 10.1177/17151635241267350 PMC11412478

[60] Schafer JJ , Gill TK , Sherman EM , McNicholl IR . ASHP guidelines on pharmacist involvement in HIV care. Am J Health Syst Pharm. 2016;73(7):468–494. 26892679 10.2146/ajhp150623

[61] Crawford ND , Myers S , Young H , Klepser D , Tung E . The role of pharmacies in the HIV prevention and care continuums: a systematic review. AIDS Behav. 2021;25(6):1819–1828. 33386509 10.1007/s10461-020-03111-w PMC8084889

[62] Guidelines on Long‐Acting Injectable Cabotegravir for HIV Prevention . WHO Guidelines Approved by the Guidelines Review Committee. Geneva; 2022. 36417549

[63] Delany‐Moretlwe S , Hughes JP , Bock P , Ouma SG , Hunidzarira P , Kalonji D , et al. Cabotegravir for the prevention of HIV‐1 in women: results from HPTN 084, a phase 3, randomised clinical trial. Lancet. 2022;399(10337):1779–1789. 35378077 10.1016/S0140-6736(22)00538-4 PMC9077443

[64] Landovitz RJ , Donnell D , Clement ME , Hanscom B , Cottle L , Coelho L , et al. Cabotegravir for HIV prevention in cisgender men and transgender women. N Engl J Med. 2021;385(7):595–608. 34379922 10.1056/NEJMoa2101016 PMC8448593

[65] Kelley CF , Acevedo‐Quiñones M , Agwu AL , Avihingsanon A , Benson P , Blumenthal J , et al. Twice‐Yearly Lenacapavir for HIV Prevention in Men and Gender‐Diverse Persons. N Engl J Med. 2025;392(13):1261–1276. 10.1056/NEJMoa2411858. 39602624

[66] Adams AJ , Klepser ME . Pharmacist prescribing models for HIV pre‐exposure and post‐exposure prophylaxis. Ann Pharmacother. 2024;58(4):434–440. 37480245 10.1177/10600280231187171

[67] Houle SK , Grindrod KA , Chatterley T , Tsuyuki RT . Paying pharmacists for patient care: a systematic review of remunerated pharmacy clinical care services. Can Pharm J (Ott). 2014;147(4):209–232. 25360148 10.1177/1715163514536678 PMC4212445

[68] Curran GM , Bauer M , Mittman B , Pyne JM , Stetler C . Effectiveness‐implementation hybrid designs: combining elements of clinical effectiveness and implementation research to enhance public health impact. Med Care. 2012;50(3):217–226. 22310560 10.1097/MLR.0b013e3182408812 PMC3731143

[69] Elnaem MH , Rosley NFF , Alhifany AA , Elrggal ME , Cheema E . Impact of pharmacist‐led interventions on medication adherence and clinical outcomes in patients with hypertension and hyperlipidemia: a scoping review of published literature. J Multidiscip Healthc. 2020;13:635–645. 32764955 10.2147/JMDH.S257273 PMC7381776

[70] Kooij MJ , Heerdink ER , van Dijk L , van Geffen EC , Belitser SV , Bouvy ML . Effects of telephone counseling intervention by pharmacists (TelCIP) on medication adherence; results of a cluster randomized trial. Front Pharmacol. 2016;7:269. 27625605 10.3389/fphar.2016.00269 PMC5003869

[71] Alfian SD , van Boven JFM , Abdulah R , Sukandar H , Denig P , Hak E . Effectiveness of a targeted and tailored pharmacist‐led intervention to improve adherence to antihypertensive drugs among patients with type 2 diabetes in Indonesia: a cluster randomised controlled trial. Br J Clin Pharmacol. 2021;87(4):2032–2042. 33085801 10.1111/bcp.14610 PMC8056734

[72] Plechschmidt J , Fietkau K , Hepp T , Dietrich P , Fischer S , Krebs S , et al. Clinical pharmacist counselling improves long‐term medication safety and patient‐reported outcomes in anti‐TNF‐treated patients with inflammatory bowel diseases: the prospective, randomized AdPhaNCED trial. Inflamm Bowel Dis. 2025;31(1):77–86. 38507608 10.1093/ibd/izae040 PMC11700895

[73] Unni EJ , Patel K , Beazer IR , Hung M . Telepharmacy during COVID‐19: a scoping review. Pharmacy (Basel). 2021;9(4):183. 34842823 10.3390/pharmacy9040183 PMC8628897

[74] Hussain A , Bowen AM . Exploring pharmacist roles in telepharmacy for chronic disease management in New York State: a qualitative inquiry into improving implementation, patient communication, and healthcare technology support. Cureus. 2024;16(6):e62982. 39044868 10.7759/cureus.62982 PMC11265808

[75] Pathak S , Blanchard CM , Moreton E , Urick BY . A systematic review of the effect of telepharmacy services in the community pharmacy setting on care quality and patient safety. J Health Care Poor Underserved. 2021;32(2):737–750. 34120974 10.1353/hpu.2021.0102

[76] Alter M , Lakhani S , Alaa A , Karki M , Riboli‐Sasco E , El‐Osta A . Investigating facilitators and barriers to the routine provision of HIV PrEP in community pharmacies in London. BMC Health Serv Res. 2025;25(1):312. 40001055 10.1186/s12913-025-12336-1 PMC11863588

[77] Bruno C , Saberi P . Pharmacists as providers of HIV pre‐exposure prophylaxis. Int J Clin Pharm. 2012;34(6):803–806. 23073703 10.1007/s11096-012-9709-0 PMC3501608

[78] Alaa A , Mujong D , Lakhani S , Alter M , El‐Osta A . Investigating the potential accessibility to HIV pre‐exposure prophylaxis via community pharmacies and sexual health clinics: a scoping review of two integrated care systems in London. BMC Health Serv Res. 2025;25(1):878. 40598347 10.1186/s12913-025-12985-2 PMC12219986

[79] Thorakkattil SA , Parakkal SA , Mohammed Salim KT , Arain S , Krishnan G , Madathil H , et al. Improving patient safety and access to healthcare: the role of pharmacist‐managed clinics in optimizing therapeutic outcomes. Explor Res Clin Soc Pharm. 2024;16:100527. 39469652 10.1016/j.rcsop.2024.100527 PMC11513600

[80] Marcum ZA , Jiang S , Bacci JL , Ruppar TM . Pharmacist‐led interventions to improve medication adherence in older adults: a meta‐analysis. J Am Geriatr Soc. 2021;69(11):3301–3311. 34287846 10.1111/jgs.17373 PMC8595553

[81] Booker C , Murphy AL , Isenor JE , Ramsey TD , Smith AJ , Bishop A , et al. Community pharmacists’ acceptance of prescribing pre‐exposure prophylaxis (PrEP) for human immunodeficiency virus (HIV). Can Pharm J. 2023;156(3):137–149. 10.1177/17151635231152218 PMC10186867 37201164

[82] Angibaud M , Jourdain M , Girard S , Rouxel L , Mouhib A , Nogueira A , et al. Involving community pharmacists in interprofessional collaboration in primary care: a systematic review. BMC Prim Care. 2024;25(1):103. 38561676 10.1186/s12875-024-02326-3 PMC10983710

[83] Paolinelli JPV , de Alencar T , Rocha KSS , Pereira ML , Dos Santos Junior GA . Implementation of clinical pharmacy services in primary health care: a scoping review. J Eval Clin Pract. 2025;31(6):e70285. 40996324 10.1111/jep.70285 PMC12462563

[84] Giannitrapani KF , Glassman PA , Vang D , McKelvey JC , Thomas Day R , Dobscha SK , et al. Expanding the role of clinical pharmacists on interdisciplinary primary care teams for chronic pain and opioid management. BMC Fam Pract. 2018;19(1):107. 29970008 10.1186/s12875-018-0783-9 PMC6031118

[85] Haberer JE , Mujugira A , Mayer KH . The future of HIV pre‐exposure prophylaxis adherence: reducing barriers and increasing opportunities. Lancet HIV. 2023;10(6):e404–e11. 37178710 10.1016/S2352-3018(23)00079-6

[86] Saberi P , Dong BJ , Johnson MO , Greenblatt RM , Cocohoba JM . The impact of HIV clinical pharmacists on HIV treatment outcomes: a systematic review. Patient Prefer Adherence. 2012;6:297–322. 22536064 10.2147/PPA.S30244 PMC3333818

