# Supplementary material for: Role of pharmacists in HIV prophylaxis: a scoping review of pharmacists’ services, interventions and outcomes
Source: J Int AIDS Soc. 2026 Feb 24;29(2):e70090. doi: 10.1002/jia2.70090 (PMC12930320; doi:10.1002/jia2.70090)
Supplement: Supplementary file 1 — Supplementary Material 1: Search strategies used in the consulted databases for the scoping review (searches conducted on May 2025). Supplementary Material 2. Excluded records with their respective reasons for exclusion during the full‐text eligibility assessment. [file JIA2-29-e70090-s001.docx]

**SUPPLEMENTARY MATERIALS**

**Supplementary Material 1. Search strategies used in the consulted databases for the scoping review (searches conducted on May 2025)**

| **Database** | **Search Strategy** |
| --- | --- |
| PubMed | #1 (Pre-Exposure Prophylaxis[Mesh Terms]) OR (Pre-Exposure Prophylaxis[Title/Abstract]) OR (Pre Exposure Prophylaxis[Title/Abstract]) OR (PrEP[Title/Abstract]) OR (Post-Exposure Prophylaxis[Mesh Terms]) OR (Post Exposure Prophylaxis[Title/Abstract]) OR (Post-Exposure Prevention[Title/Abstract]) OR (PEP[Title/Abstract]) OR (HIV Prevention[Title/Abstract]) OR (HIV Prophylaxis[Title/Abstract]) #2 (Pharmacists[MeSH Terms]) OR (Pharmacist[Title/Abstract]) OR (Clinical Pharmacists[Title/Abstract]) OR (Clinical Pharmacist[Title/Abstract]) OR (Community Pharmacists[Title/Abstract]) OR (Community Pharmacist[Title/Abstract]) OR (Pharmacies[MeSH Terms]) OR (Community Pharmacy[Title/Abstract]) OR (Community Pharmacies[Title/Abstract]) OR (Pharmaceutical Services[Mesh Terms]) OR (Pharmaceutic Services[Title/Abstract]) OR (Pharmaceutic Service[Title/Abstract]) OR (Pharmaceutical Service[Title/Abstract]) OR (Pharmacy Services[Title/Abstract]) OR (Pharmacy Service[Title/Abstract]) OR (Pharmaceutical Care[Title/Abstract]) #1 AND #2 |
| Embase | #1 'pre-exposure prophylaxis'/exp OR 'post exposure prophylaxis'/exp OR 'pre-exposure prophylaxis':ab,ti OR 'pre exposure prophylaxis':ab,ti OR 'PrEP':ab,ti OR 'post-exposure prophylaxis':ab,ti OR 'post exposure prophylaxis':ab,ti OR 'PEP':ab,ti OR 'HIV prevention':ab,ti OR 'HIV prophylaxis':ab,ti #2 'pharmacist'/exp OR 'pharmacy practice'/exp OR 'clinical pharmacist'/exp OR 'community pharmacist'/exp OR 'pharmacist':ab,ti OR 'clinical pharmacists':ab,ti OR 'clinical pharmacist':ab,ti OR 'community pharmacists':ab,ti OR 'community pharmacist':ab,ti OR 'pharmacy':ab,ti OR 'pharmaceutical services':ab,ti OR 'pharmaceutic services':ab,ti OR 'pharmacy services':ab,ti OR 'pharmacy service':ab,ti OR 'pharmaceutical care':ab,ti #3 #1 AND #2 |
| Scopus | (TITLE-ABS-KEY("pre-exposure prophylaxis") OR TITLE-ABS-KEY("pre exposure prophylaxis") OR TITLE-ABS-KEY("PrEP") OR TITLE-ABS-KEY("post-exposure prophylaxis") OR TITLE-ABS-KEY("post exposure prophylaxis") OR TITLE-ABS-KEY("PEP") OR TITLE-ABS-KEY("HIV prevention") OR TITLE-ABS-KEY("HIV prophylaxis")) AND (TITLE-ABS-KEY("pharmacist") OR TITLE-ABS-KEY("pharmacy practice") OR TITLE-ABS-KEY("clinical pharmacists") OR TITLE-ABS-KEY("community pharmacists") OR TITLE-ABS-KEY("pharmacy") OR TITLE-ABS-KEY("pharmaceutical services") OR TITLE-ABS-KEY("pharmacy services") OR TITLE-ABS-KEY("pharmaceutical care")) |
| LILACS | ((MH:pre-exposure prophylaxis) OR (pre-exposure prophylaxis) OR (MH:post-exposure prophylaxis) OR (post-exposure prophylaxis) OR (PrEP) OR (PEP) OR (HIV prevention) OR (HIV prophylaxis)) AND ((MH:pharmacist) OR (pharmacist*) OR (clinical pharmacist) OR (community pharmacist) OR (MH:pharmaceutical services) OR (pharmaceutical services)) |
| Google Scholar | (“pre-exposure prophylaxis” OR “post-exposure prophylaxis” OR “PrEP” OR “PEP” OR “HIV prevention” OR “HIV prophylaxis”) AND (“pharmacist” OR “clinical pharmacist” OR “community pharmacist” OR “pharmaceutical services”) |

**Supplementary Table 2. Excluded records with their respective reasons for exclusion during the full-text eligibility assessment**

| **Author (year)** | **Title** | **Reason for exclusion** |
| --- | --- | --- |
| Cockerill RW, et al. (1996) | Pharmacies and their role in the prevention of HIV/AIDS | Study assessed perception, preference or willingness |
| Sheridan J, et al. (1997) | Drug Misuse and HIV Prevention: Attitudes and  Practices of Community Pharmacists with Respect  to two London Family Health Services Authorities | Clinical activities of pharmacists were not described |
| Sheridan J, et al. (1997) | HIV prevention and drug treatment services for drug misusers: a national study of community pharmacists' attitudes and their involvement in service specific training | Clinical activities of pharmacists were not described |
| Gleghorn AA, et al. (1998) | Pharmacists' attitudes about pharmacy sale of needles/syringes and needle exchange programs in a city without needle/syringe prescription laws | Clinical interventions of pharmacists were not described |
| Hensic LA, et al. (2010) | Post-exposure prophylaxis regimens for occupational exposures to HIV | Incorrect publication type |
| Zaller N, et al. (2010) | Pharmacist and pharmacy staff experiences with non-prescription (NP) sale of syringes and attitudes toward providing HIV prevention services for injection drug users (IDUs) in Providence, RI | Study assessed perception, preference or willingness |
| Fuller CM, et al. (2013) | Pharmacist and clinician attitudes toward use of a web- application to support co-management of post-exposure prophylaxis patients | Study assessed perception, preference or willingness |
| Smati J, et al. (2015) | Who does receive the virus post-exposure prophylaxis? | Incorrect publication type |
| Ferrell KW, et al. (2015) | Role of medication therapy management in preexposure prophylaxis therapy for HIV prevention | Incorrect publication type |
| Lewis CF, et al. (2015) | Pharmacy-randomized intervention delivering HIV prevention services during the syringe sale to people who inject drugs in New York City | Clinical interventions of pharmacists were not described |
| Sawkin M, et al. (2016) | Development of a pharmacist-led human immunodeficiency virus pre-exposure prophylaxis clinic at an urban community clinic | Incorrect publication type |
| Cayre F, et al. (2016) | Advantages of incremental dispensing of post exposure prophylaxis (PEP) drugs and comparison of two care pathways (CP) | Incorrect publication type |
| Chiampas T, et al. (2016) | Description of collaboration between an interdisciplinary human immunodeficiency virus (HIV) clinic | Incorrect publication type |
| Ragland A, et al. (2017) | Knowledge and attitudes of hiv pre-exposure prophylaxis among community pharmacists in the greater kansas city area | Incorrect publication type |
| Tung E, et al. (2017) | Feasibility of a pharmacist-run HIV PrEP clinic in a community pharmacy setting | Incorrect publication type |
| Not reported (clinical trial registry) | A Brief, Pragmatic Experiment to Determine the Impact of a Public-health-partnered Tele-pharmacist Model for PrEP Delivery in a Rural State | Incorrect publication type |
| Miller M, et al. (2017) | Implementation of a pharmacist-led pre-exposure HIV prophylaxis (PrEP) Clinic | Incorrect publication type |
| Ryan K, et al. (2018) | The next step in PrEP: Evaluating outcomes of a pharmacist-run HIV pre-exposure prophylaxis (PrEP) clinic | Incorrect publication type |
| Hoth A, et al. (2018) | Iowa TelePrEP: Preliminary experience with a public health-partnered, telemedical PrEP delivery model in a rural state | Incorrect publication type |
| Bares S, et al. (2018) | Acceptability and Feasibility of a Pharmacist-led Pre-exposure Prophylaxis Program in the Midwestern United States | Incorrect publication type |
| Smith BL, et al. (2019) | A Pharmacist-led PrEP Program at the Epicenter of the HIV Epidemic in Atlanta; Our Experience | Incorrect publication type |
| Grossman KH (2020) | Improving patient access to HIV post-exposure prophylaxis with pharmacist involvement | Incorrect publication type |
| Whelchel K, et al. (2020) | Extended adherence and persistence to HIV prep in a multidisciplinary prep clinic | Incorrect publication type |
| Reno H, et al. (2020) | PrEP and follow up STD testing in a Midwest public sexual health clinic using a pharmacy co-management model | Incorrect publication type |
| Grimshaw C, et al. (2020) | Delivery of oral HIV pre-exposure prophylaxis for people who inject drugs and are at risk of sexual exposure to HIV during an outbreak | Incorrect publication type |
| Nisly NL, et al. (2020) | Changing healthcare inequalities by offering AV new model for healthcare delivery: pharmacist delivered prep for people of all genders | Incorrect publication type |
| Crawford ND, et al. (2020) | Pharmacy-based pre-exposure prophylaxis support among pharmacists and men who have sex with men | Clinical interventions of pharmacists were not described |
| Koester KA, et al. (2020) | Attitudes about community pharmacy access to HIV prevention medications in California | Clinical interventions of pharmacists were not described |
| Zhu V, et al. (2020) | Patient perception of community pharmacists prescribing pre-exposure prophylaxis for HIV prevention | Study assessed perception, preference or willingness |
| Doblecki-Lewis S, et al. (2020) | Implementation of mobile prep, STI, and HIV-prevention services in South Florida | Incorrect publication type |
| Lowrey K, et al. (2020) | Improvement in HIV screening follow-up with emergency department pharmacist dispensing of post-exposure prophylaxis | Incorrect publication type |
| Ortblad KF, et al. (2020) | Design of a care pathway for pharmacy-based PrEP delivery in Kenya: results from a collaborative stakeholder consultation | Study assessed perception, preference or willingness |
| Wagner G, et al. (2021) | Predictors of PrEP uptake in a sexual health clinic with immediate PrEP initiation | Incorrect publication type |
| Bouetard L, et al. (2021) | Knowledge, experience and perception of pharmacists in Ile-de- France regarding HIV pre-exposure prophylaxis (PrEP) | Incorrect publication type |
| Yacout H, et al. (2021) | PrEP Adherence and Discontinuation at a Pharmacy-Supported PrEP Program in Atlanta, GA | Incorrect publication type |
| Yang E, et al. (2021) | Evaluation of Patients at High Risk of Acquiring Human Immunodeficiency Virus Prior to Implementation of Pharmacists in the Interdisciplinary Pre-Exposure Prophylaxis Service | Incorrect publication type |
| Jacomet C, et al. (2022) | To what extent do people living with HIV, people on pre-exposure prophylaxis, doctors and pharmacists endorse 90-day dispensing of antiretroviral therapy in France? | Clinical interventions of pharmacists were not described |
| Hiner D, et al. (2022) | Pharmacist Impact on Pre-Exposure Prophylaxis Uptake in High-Risk Populations | Incorrect publication type |
| Cernasev A, et al. (2022) | Tennessee Pharmacists’ Opinions on Barriers and Facilitators to Initiate PrEP: A Qualitative Study | Study assessed perception, preference or willingness |
| Whelchel K, et al. (2022) | Pharmacy model impact on HIV PrEP persistence | Incorrect publication type |
| Barner A, et al. (2022) | Failing to PrEPare is PrEParing to Fail: Advocating for PWID PrEP Inclusion | Incorrect publication type |
| Ortblad K, et al. (2022) | Pharmacy-based PrEP initiation and continuation in Kenya: findings from a pilot study | Incorrect publication type |
| Vila Gallego, C, et al. (2022) | Human immunodeficiency virus pre-exposure prophylaxis: analysis, follow-up and pandemic effect | Incorrect publication type |
| Aramendi IB, et al. (2022) | Description and follow-up of the use of emtricitabin/tenofovir for HIV pre-exposure prophylaxis | Incorrect publication type |
| Kherghehpoush S, et al. (2023) | The pharmacist's role in screening patients experiencing homelessness for HIV and Hepatitis C | Incorrect publication type |
| Roche S, et al. (2023) | Pharmacy-based PrEP delivery in Kenya: findings from a pilot study extension | Incorrect publication type |
| Kleinmann W, et al. (2023) | High rates of HIV PrEP prescriptions after standardized counseling of high-risk pregnant patients | Incorrect publication type |
| Cerulli J, et al. (2023) | Achieving quality pharmacy care for HIV post exposure prophylaxis (PEP) therapy | Incorrect publication type |
| Kuo A, et al. (2023) | Costs of providing pharmacy-initiated PrEP in Kenya: findings from a pilot study | Incorrect publication type |
| Dourado I, et al. (2023) | Interdisciplinarity in HIV prevention research: the experience of the PrEP1519 study protocol among adolescent MSM and TGW in Brazil | Clinical interventions of pharmacists were not described |
| MacDonald CB, et al. (2023) | Target users’ acceptance of a pharmacist-led prescribing service for pre-exposure prophylaxis (PrEP) for human immunodeficiency virus (HIV) | Study assessed perception, preference or willingness |
| Asewe M, et al. (2024) | The Acceptability of Pharmacy-delivered Prep in Kenya: Provider and Client Perceptions | Incorrect publication type |
| Babatunde YO, et al. (2024) | Acceptability and feasibility to access PrEP services through a pharmacy-based delivery model among adolescents and young adults in Lagos, Nigeria: Lessons learned from a community-engaged approach | Incorrect publication type |
| Nakate V, et al. (2024) | Acceptability of and willingness to use HIV pre-exposure prophylaxis in community Pharmacy users in Uganda | Incorrect publication type |
| Padilla E, et al. (2024) | Improving adherence to HIV PREP via a PBM- driven educational intervention | Incorrect publication type |
| Herron G, et al. (2024) | Assessment of PrEP and PEP furnishing in San Francisco Bay Area pharmacies: An observational cross-sectional study | Clinical interventions of pharmacists were not described |
| Washington TA, et al. (2024) | Recommendations from Black and Latinx sexual minority males to include pharmacists to increase greater accessibility and OnlyFans stars to promote uptake of injectable PrEP | Study assessed perception, preference or willingness |
| Ekwunife OI, et al. (2024) | Collaborative design of a care pathway for pharmacy-based PrEP delivery in Nigeria: insights from stakeholder consultation | Study assessed perception, preference or willingness |
| Harrison C, et al. (2024) | A qualitative exploration of pharmacists and customers barriers and facilitators to community pharmacy PrEP delivery using the COM-B model of behaviour change | Incorrect publication type |
| Adams AJ, et al. (2024) | Pharmacist Prescribing Models for HIV Pre-exposure and Post-exposure Prophylaxis | Incorrect publication type |
| Mercer KJ, et al. (2024) | Expanding expedited partner therapy and HIV prophylaxis in the emergency department | Incorrect publication type |
| Roche S, et al. (2025) | Implementing long-acting injectable HIV pre-exposure prophylaxis services at private pharmacies in Kenya: Client, pharmacy provider, and key stakeholder perspectives on potential challenges and opportunities | Incorrect publication type |
| Chen Y, et al. (2025) | Incremental cost of pre- and post-exposure prophylaxis service provision via an online pharmacy in Kenya | Clinical interventions of pharmacists were not described |
| Harrison C, et al. (2025) | Qualitative exploration of the barriers and facilitators to community pharmacy PrEP delivery for UK pharmacists and underserved community members using the COM-B model of behaviour change | Study assessed perception, preference or willingness |
